# Effects of Synbiotic Supplement on Human Gut Microbiota, Body Composition and Weight Loss in Obesity

**DOI:** 10.3390/nu12010222

**Published:** 2020-01-15

**Authors:** Igor N. Sergeev, Thamer Aljutaily, Gemma Walton, Eduardo Huarte

**Affiliations:** 1 Department of Health and Nutritional Sciences, South Dakota State University, Brookings, SD 57007, USA; thamer.aljutaily@jacks.sdstate.edu (T.A.); eduardohuarte@yahoo.es (E.H.); 2 Department of Food and Nutritional Sciences, University of Reading, Reading RG6 6AP, UK; g.e.walton@reading.ac.uk

**Keywords:** synbiotic, prebiotic, probiotic, gut microbiota, obesity, weight loss, body composition, obesity biomarkers

## Abstract

Targeting gut microbiota with synbiotics (probiotic supplements containing prebiotic components) is emerging as a promising intervention in the comprehensive nutritional approach to reducing obesity. Weight loss resulting from low-carbohydrate high-protein diets can be significant but has also been linked to potentially negative health effects due to increased bacterial fermentation of undigested protein within the colon and subsequent changes in gut microbiota composition. Correcting obesity-induced disruption of gut microbiota with synbiotics can be more effective than supplementation with probiotics alone because prebiotic components of synbiotics support the growth and survival of positive bacteria therein. The purpose of this placebo-controlled intervention clinical trial was to evaluate the effects of a synbiotic supplement on the composition, richness and diversity of gut microbiota and associations of microbial species with body composition parameters and biomarkers of obesity in human subjects participating in a weight loss program. The probiotic component of the synbiotic used in the study contained *Lactobacillus acidophilus*, *Bifidobacterium lactis*, *Bifidobacterium longum*, and *Bifidobacterium bifidum* and the prebiotic component was a galactooligosaccharide mixture. The results showed no statistically significant differences in body composition (body mass, BMI, body fat mass, body fat percentage, body lean mass, and bone mineral content) between the placebo and synbiotic groups at the end of the clinical trial (3-month intervention, 20 human subjects participating in weight loss intervention based on a low-carbohydrate, high-protein, reduced energy diet). Synbiotic supplementation increased the abundance of gut bacteria associated with positive health effects, especially *Bifidobacterium* and *Lactobacillus*, and it also appeared to increase the gut microbiota richness. A decreasing trend in the gut microbiota diversity in the placebo and synbiotic groups was observed at the end of trial, which may imply the effect of the high-protein low-carbohydrate diet used in the weight loss program. Regression analysis performed to correlate abundance of species following supplementation with body composition parameters and biomarkers of obesity found an association between a decrease over time in blood glucose and an increase in *Lactobacillus* abundance, particularly in the synbiotic group. However, the decrease over time in body mass, BMI, waist circumstance, and body fat mass was associated with a decrease in *Bifidobacterium* abundance. The results obtained support the conclusion that synbiotic supplement used in this clinical trial modulates human gut microbiota by increasing abundance of potentially beneficial microbial species.

## 1. Introduction

The gut microbiota appears to play a role in the pathogenesis of obesity and associated diseases [1,2]. This community can contribute to the development of obesity primarily by influencing dietary energy intake and intestinal absorption of nutrients [3,4], but it can also provide the human host with benefits besides energy extraction, including a reduction of low grade chronic inflammation associated with obesity and metabolic syndrome [5,6]. Therefore, gut microbiota may be considered a promising target in the comprehensive dietary approach to the prevention and treatment of obesity, including weight loss and weight maintenance [7,8].

It is important to note that high-protein and low-carbohydrate diets, which are often successfully used for weight loss, have been associated with a decrease in bacteria considered beneficial to health [9,10,11]. These diets have also been found to induce protein fermentation by gut microbiota with formation of metabolic byproducts [12,13,14], which can trigger inflammation in the colon [15]. Furthermore, high levels of protein fermentation by gut bacteria have been associated with increased genotoxicity, which may be associated with bowel cancers [16], indicating that a less toxic gut microbiota is critical for wellbeing of the host [17,18].

Dietary intervention with probiotics, prebiotics or synbiotics (which combine probiotic and prebiotic components) aimed at correcting disruption of the gut microbiota observed in obesity or following imbalanced diets may provide health benefits by facilitating weight loss and maintenance [19,20]. Recent human and animal studies have suggested that probiotics can promote weight loss in obesity [21,22], but studies on the role of synbiotics in obesity are very limited [23,24] and further studies are warranted [23].

The objective of this placebo-controlled intervention clinical trial was to evaluate the effects of a synbiotic supplement containing *Bifidobacterium* and *Lactobacillus* strains on the human gut microbiota in relation to changes in body composition and metabolic biomarkers in obesity. The results obtained and bioinformatic analysis support the conclusion that the synbiotic supplement used in this study modulates the human gut microbiota by increasing the abundance of beneficial microbial genera.

## 2. Materials and Methods

### 2.1. Study Participants, Clinical Trial Design and Prebiotic Supplement

The participants of the study were enrolled in the weight loss program (Profile by Sanford Health, Sioux Falls, SD). Twenty new weight-loss participants, male and female, were recruited for the study and randomly assigned to the placebo (control) or synbiotic (treatment) group. Those enrolled were initially overweight/obese and had a mean BMI of 33.5 kg/m^2^. The placebo group (*n* = 10) followed the weight loss program eating plan (a low-carbohydrate high-protein dietary pattern with reduced energy intake). A typical daily meal plan included 104 g protein, 68–80 g carbohydrates, 36.5–39.0 g fat, and 26–30 g fiber (4100–4160 kJ). These were the dietary recommendations and we did not track adherence in a way that provided macronutrient and micronutrient composition of what was actually consumed. The synbiotic group (*n* = 10) was on the same diet plan, but additionally received a synbiotic (probiotic plus prebiotic) supplement daily for 3 months. The control group received the placebo supplement similar in appearance and of the same energy content as the synbiotic supplement. Human subjects with conditions that may impact gut microbiota (gastrointestinal, autoimmune, and metabolic diseases and medications, particularly antibiotics) were not included in the trial. All subjects gave their informed consent for participating in the study. The study was approved by the SDSU Institutional Research Board (Approval number: IRB-1604005). The clinical trial has been registered at ClinicalTrials.gov (NCT number: 03123510).

The probiotic component of the synbiotic used in the study contained a blend (one capsule contained 69 mg or 15 × 10^9^ CFU) of proprietary strains of *Lactobacillus acidophilus* DDS-1*, Bifidobacterium lactis* UABla-12, *Bifidobacterium longum* UABl-14, and *Bifidobacterium bifidum* UABb-10. The probiotic supplement was produced by UAS Labs (Wausau, WI). The prebiotic component was a *trans*-galactooligosaccharide (GOS) mixture at a dose of 5.5 g/d (2.75 g GOS and the remainder simple sugars) produced by Clasado BioSciences Ltd. (St. Helier, Jersey, UK) [25].

### 2.2. Body Composition and Metabolic Parameters

Body mass index (BMI) was calculated using body weight and height measured with bare feet and in minimal clothing using a stadiometer and an electronic scale. Body composition parameters (body fat mass and percentage, body lean mass, and bone mineral content) were acquired before and after 3 months of synbiotic intervention by dual-energy X-ray absorptiometry (DXA) using a whole-body scanner (Hologic APEX, Bedford, MA, USA) [26]. Whole-body images were obtained and analyzed by a technologist certified as a Certified Bone Densitometry Technologist by the International Society of Clinical Densitometry. Phantom scans were performed before participant testing as an independent assessment of system calibration, and quality control data were plotted and reviewed periodically. Seven human subjects in the placebo group and 8 human subjects in the synbiotic group completed DXA scans. The A1CNow^+^ test (MDSS GmbH, Hannover, Germany/Polymer Technology Systems, Inc., Indianapolis, IN, USA) was used for quantitative measurement of the percent of glycated hemoglobin (%A1C) in the capillary blood (fingerstick) samples.

### 2.3. Microbial DNA Extraction and the 16S rRNA Gene Sequencing

Fecal samples from the participants were obtained before and after the synbiotic or placebo intervention no more than 24 h prior to the study visit. Samples were collected using the OMNIgene-GUT stool/feces collection kit (OMR-200, DNA Genotek, Ottawa, ON, Canada). Forty fecal specimens from unique participants were sent to DNA Genotek for the microbiome analysis. DNA was extracted and quantified and library preparation was performed with Illumina’s NexteraXT protocol. Aliquots of each sample were extracted using an PowerMag microbial DNA isolation kit (MO BIO Laboratories, Carslbad, CA, USA) optimized for the KingFisher Flex automated extraction platform (ThermoFisher, Pittsburgh, PA, USA). A bead-beating step with glass beads was used to maximize recovery of DNA from low-abundance and difficult-to-lyse microorganisms. The concentration of extracted DNA was measured using a Qubit Fluorometer (Invitrogen, Carslbad, CA, USA) and sample purity was confirmed spectrophotometrically by measuring the A260/A280 ratio.

For DNA sequencing, Illumina sequencing adapters and dual-index barcodes (Nextera XT indices) were added to the amplicon target via polymerase chain reaction (PCR) amplification. 16S sequencing (2 × 300 bp PE V3-V4) was performed on Illumina’s MiSeq platform. Amplicon sequencing was performed to a target depth of 30,000 reads per sample. Paired-end reads from each sample were merged, screened for length and filtered for quality using DNA Genotek’s proprietary 16S pre-processing workflow. Read merging and quality filtering was performed on the raw sequencing reads to eliminate any sequencing artifacts and low-quality reads. Complete quality metrics including library quantification and sequencing run quality control are presented in the Supplemental Materials (Figure S1 and Table S1). 

### 2.4. Taxonomic Classification and Bioinformatics Analysis

A curated reference taxonomic database was used to assign a taxonomic classification to the sequencing reads. High-quality sequences were aligned to the curated reference database at 97% similarity using the NINJA-OPS algorithm, version 1.5.1 [27]. At 97% sequence identity, each operational taxonomic unit (OTU) represents a genetically unique group of biological organisms. These OTUs were then assigned a curated taxonomic label based on the SILVA taxonomic database, version 123 [28]. The relative abundance of all taxa at the phylum and genus levels were plotted to visualize sample-specific classifications. All samples were rarefied to an even depth of 25,000 classified sequences per sample or more to eliminate effects of variance in sequencing depth. Samples with more than 25,000 classified sequences per sample were included in the rarefied OTU table and downstream analyses, thus allowing rarefication of the samples to 52,150 read pairs/sample, i.e., the read count of the sample with the fewest reads (see Table S1). 

Alpha diversity metrics (observed OTUs, Shannon index, and Chao1 diversity) were calculated on the rarefied OTU table using the alpha_rarefaction.py workflow in QIIME 1.9.1 [29]. Beta diversity metrics (weighted and unweighted UniFrac distances) were calculated on the rarefied OTU table with the beta_diversity.py workflow in QIIME 1.9.1 and Bray-Curtis dissimilarity index was calculated on the species level summarization of the rarefied OTU table. Differences between groups were estimated using Permutational Multivariate Analysis of Variance (PerMANOVA; adonis function in the vegan R package). Principal Coordinates Analysis (PCoA) was applied to each beta diversity distance matrix using the dudi.pco function from the R package made4 (version 1.48.0). The first two major axes were plotted (R package ggplot2 version 2.2.1) and the percentage of variance explained by each axis was indicated.

### 2.5. Statistical Analysis

A one-way ANOVA with independent samples *t*-test was used for group comparison of the body composition and metabolic parameters (SPSS Statistics, v. 25). The results were expressed as mean ± SD, and mean differences were considered significant at *p* < 0.05. Significant differences in alpha diversity between groups were determined using estimated marginal means analysis applied to linear mixed model, built with alpha diversity as the response variable, the treatment groups and time points as the predictor variables, and subject number as a random variable. Significant differences in beta diversity between groups were determined using PerMANOVA with beta diversity as the response variable and the treatment groups and time points as predictor variables. Statistical analyses of diversity metrics were performed using R version 3.3.2 (R Core Team, 2015). 

Associations between relative abundance of gut bacteria, body composition and metabolic parameters were calculated using Pearson’s linear correlation coefficient. Regression analysis to correlate microbial abundance of species/genera present in the synbiotic supplement (*Bifidobacterium* and *Lactobacillus*) with body composition parameters and biomarkers of obesity were performed by applying ANOVA to a mixed linear model built with the percent abundance of microbe of interest as the response variable and the interaction between the specific parameter (gender, age, body mass, weight circumstance, BMI, body fat mass, body fat percentage, lean mass, bone mineral content, or HbA1C), treatment groups (placebo or synbiotic) and time points (beginning or end of trial) as predictor variables, with subject number as random variable. The Bonferroni correction method was used for multiple testing. Software versions used for data analyses are provided in Supplemental Table S2.

## 3. Results

In this placebo-controlled intervention clinical trial, the effects of the synbiotic supplement on richness and diversity of gut microbiota and associations of microbial species with measurements of body composition and biomarkers of obesity were evaluated in human subjects participating in a weight loss program. Twenty participants were recruited in the study (10 in the placebo (control) group and 10 in the synbiotic (treatment) group). The average BMI of the study participants was 33.5 kg/m^2^ and the average age was 47.4 years. The majority of participants were female (80% in the placebo group and 70% in the synbiotic group).

Participants were enrolled in the weight loss program at the beginning of the study and followed a low-carbohydrate, high-protein, reduced-energy intake eating plan. The probiotic component of the synbiotic used in the study contained *Bifidobacterium* spp. and *Lactobacillus acidophilus* strains, and the prebiotic component was a *trans*-galactooligosaccharide mixture. Blood and fecal samples were collected and body composition and metabolic parameters measured at the beginning and end of the three-month intervention trial. Seven human subjects in the placebo group and eight human subjects in the synbiotic group had body composition parameters measured using DXA. No participants dropped out of the study during the intervention period.

### 3.1. Body Composition and Metabolic Parameters

The results obtained indicate that there were no statistically significant differences in the body composition parameters (body mass, waist circumstance, BMI, body fat mass, body fat percentage, body lean mass, bone mineral content (as measured by DXA) and obesity-related biomarkers (blood glucose, as measured by HbA1C levels)) between the placebo and synbiotic groups at the end of the clinical trial (three-month synbiotic intervention) (Table 1). Body mass, waist circumference, BMI, fat mass, fat percentage, and glucose level significantly decreased or demonstrated a decreasing trend in the placebo and synbiotic groups at the end of the trial (participants in both the placebo and synbiotic groups were enrolled in the weight loss program). The decrease in HbA1C percentage at the end of trial was statistically significant in the synbiotic group, but not in the placebo group. Individual body composition parameters, including the DXA scan measurements, are presented in Supplemental Table S3.

The findings obtained demonstrate that the low-carbohydrate, high-protein, decreased-energy diet is effective for weight loss and normalizing obesity-related metabolic parameters (blood glucose), but they do not support the conclusion that the synbiotic supplement used in the study has a significant impact on body mass and body composition of human subjects participating in this weight loss program.

### 3.2. Gut Microbiota

**Figure 1:**
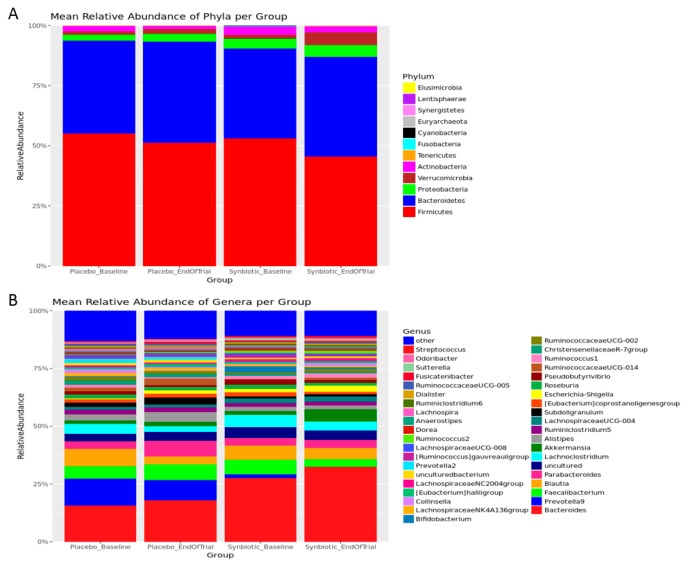
Mean relative abundance (%) of phyla (**A**) and genera (**B**) by the treatment groups and time points.

To characterize effects of the synbiotic supplement on gut microbiota of the study participants, fecal samples were obtained before and after the synbiotic intervention, gene sequence analysis was performed, and individual variations as well as group differences of gut microbiota were compared. All samples underwent taxonomic classification and were included in the complete OTU table (supplemental Table S4), however, those with fewer than 25,000 classified sequences per sample were excluded from further analysis. Remaining samples were rarefied to a depth of 52,150 sequence reads per sample. Raw read counts per sample, quality of filtered read counts per sample, and sequence quality metrics per sequencing run are provided in the Supplemental Materials (see Figure S1 and Table S1). The relative abundance of all taxa at the phylum, genus, and species levels were plotted to visualize broad taxonomic differences by treatment groups and time points with a percentage of each number in all sequencing reads (Figure 1 and Figure S2). In addition, the relative abundance of phyla, genera, and species per sample were plotted (supplemental Figure S3A–C). 

**Figure 2:**
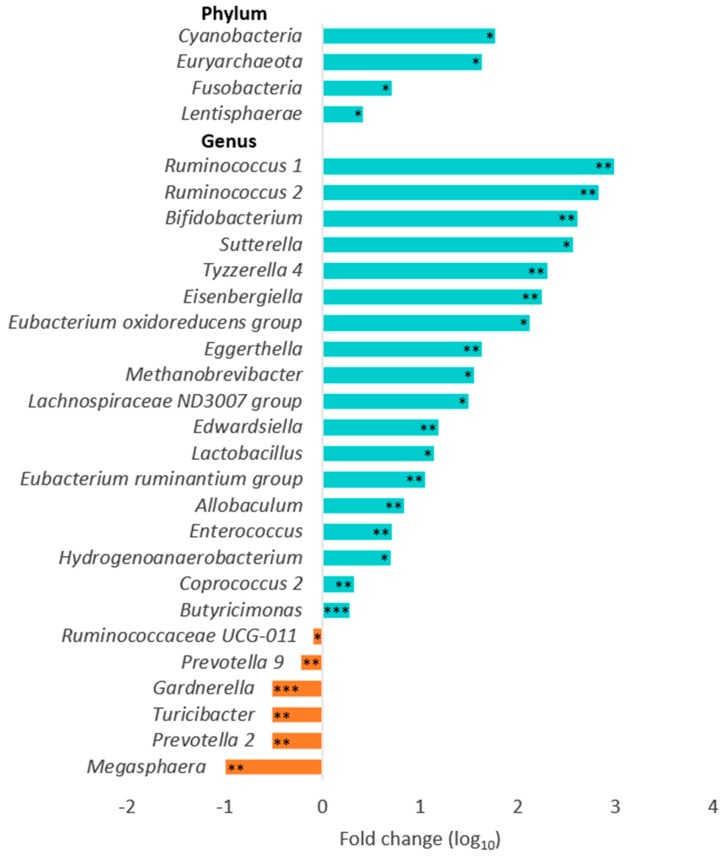
Changes in the relative microbial abundance in the gut after synbiotic intervention. * *p* < 0.05, ** *p* < 0.01, *** *p* < 0.001, as compared with the placebo group at the end of the trial.

The data obtained confirm that *Firmicutes* and *Bacteroidetes* were the two most abundant bacterial phyla in the gut (see Figure 1A) and *Bacteroides* was the most abundant genus (see Figure 1B). The synbiotic supplementation induced statistically significant alterations in the composition of the gut microbiota at the end of trial, as compared with the placebo group (Figure 2). All data remained significant after adjusting for multiple testing (supplemental Table S5). At the phylum level, increases in relative abundance of *Cyanobacteria, Euryarchaeota, Fusobacteria,* and *Lentisphaerae* were observed following synbiotic intervention. At the genus level, relative abundance of *Ruminococcus, Bifidobacterium, Sutterella, Tyzzerella, Eisenbergiella, Eubacterium, Eggerthella, Methanobrevibacter, Lachnospiraceae, Edwardsiella, Lactobacillus, Allobaculum, Enterococcus, Hydrogenoanaerobacterium, Coprococcus,* and *Butyricimonas* were significantly higher. The relative abundance of *Ruminococcaceae, Prevotella, Gardnerella*, *Turicibacter,* and *Megasphaera* at the end of trial was significantly lower in the synbiotic group, as compared with the placebo group. These results indicate that the synbiotic supplement used in the study modified the relative abundance of gut bacteria, some of which can be associated with health benefits (particularly, significantly increased abundance of *Bifidobacterium* and *Lactobacillus*).

**Figure 3:**
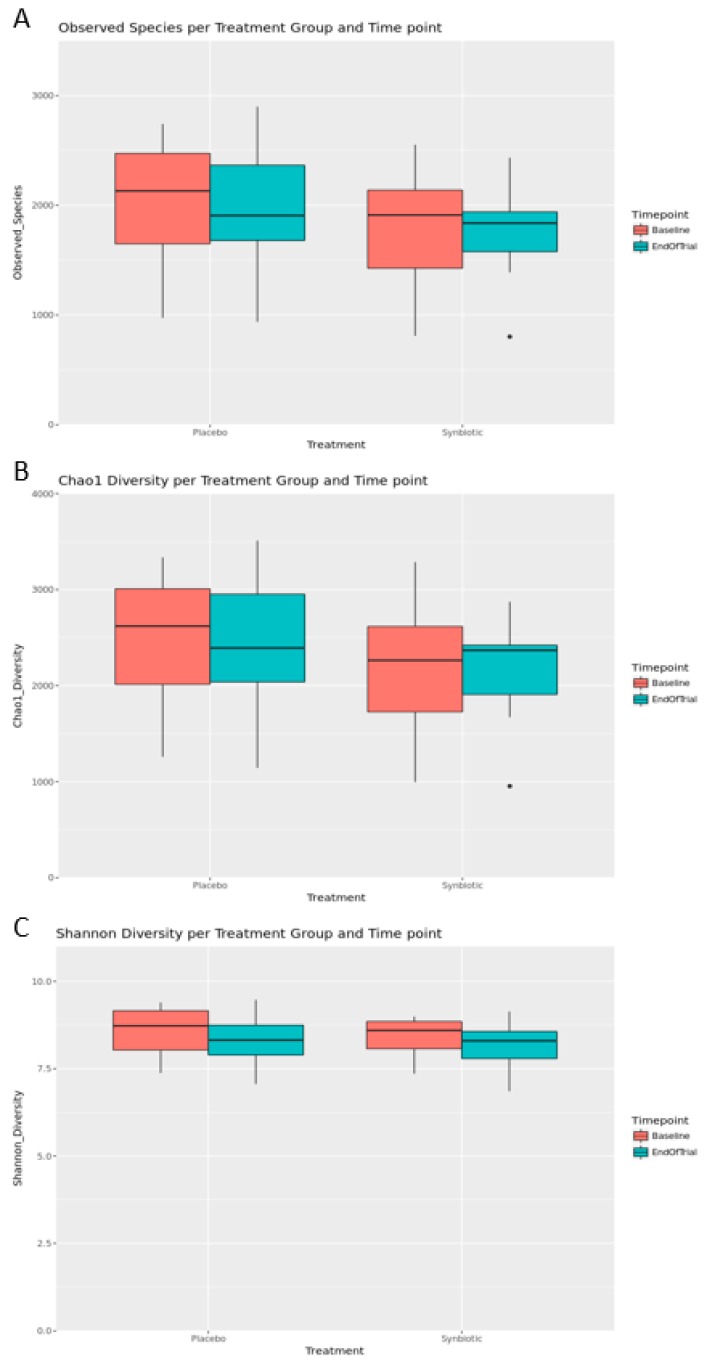
Observed species (**A**), Chao1 diversity (**B**) and Shannon diversity (**C**) plotted by the treatment group and time point. The box spans the first and third quartiles. A horizontal line marks the median and the whiskers represent ±1.5 times the interquartile range. Outliers (panels **A** and **B**) are marked as individual points. Significant differences between groups were determined using the estimated marginal means analysis applied to linear mixed model, which was built with alpha diversity as the response variable, the treatment group and time points as predictor variables, and subject number as a random variable.

Alpha diversity metrics were used to measure species richness and evenness (similar abundance) in the groups (Figure 3 and Table 2). The number of OTUs, the Chao1 estimator (a measure of community richness) [29], and the Shannon Index (a measure of richness and evenness or entropy) [30] were calculated. The data analysis showed that there were no significant differences in alpha diversity metrics between treatment groups and time points (Figure 3B). The Shannon index pointed to a decreasing trend in microbial diversity at the end of trial in both the placebo and synbiotic groups (Figure 3C). These data suggest that the observed decrease in microbial diversity in the placebo and synbiotic groups at the end of trial implies involvement of other factors, probably, the effect of the high-protein, low-carbohydrate, energy-restricted diet used in this weight loss program.

Beta diversity metrics were used to compare differences in the community composition of two different samples. Bray–Curtis dissimilarity was used to compare the abundance of each OTU between two samples to give a metric between 0 and 1; weighted UniFrac distance, which is a dissimilarity metric that uses the phylogenetic distribution of the OTUs in a sample together with the abundance of those OTUs to measure the distance between two samples; and unweighted UniFrac distance, which also measures the phylogenetic distribution of the OTUs in a sample, but relies only on the presence/absence data instead of abundance data [30]. An assessment of the distances within and between time points and groups did not reveal significant changes in the community structure (Table 3).

**Figure 4:**
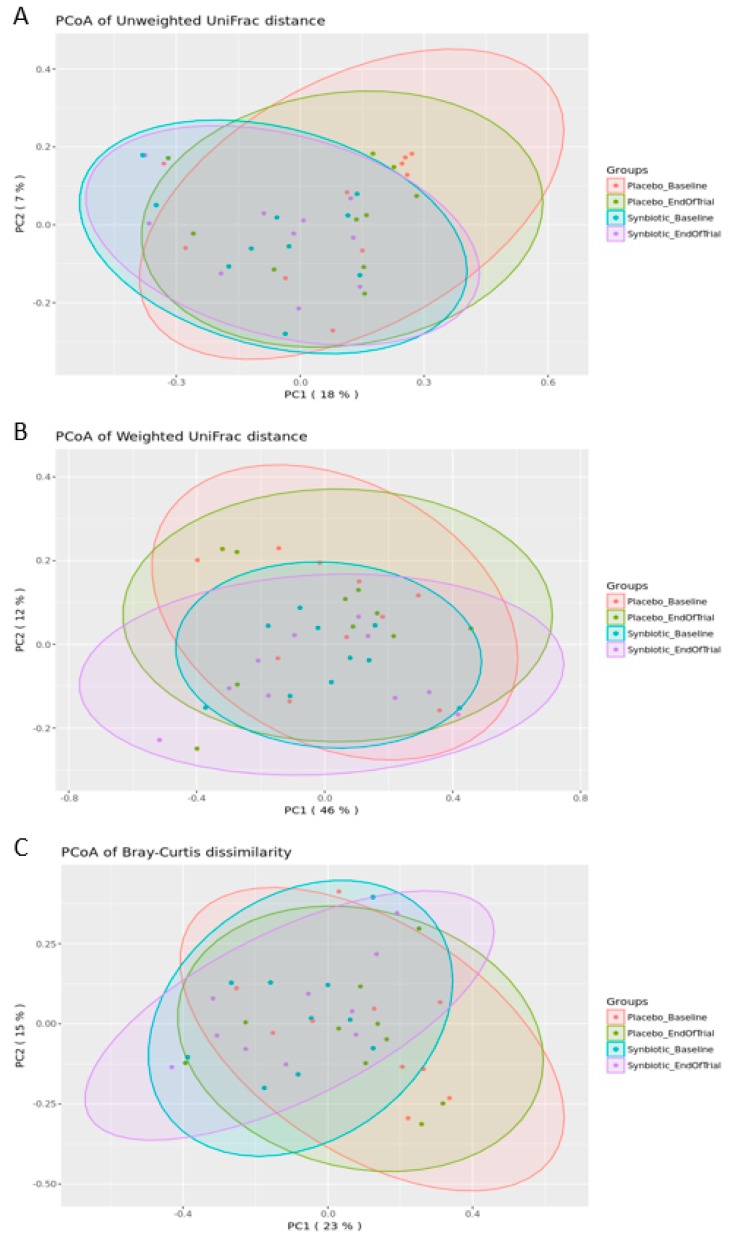
Principal Coordinates Analysis (PCoA) of unweighted UniFrac (**A**), weighted UniFrac (**B**) and Bray–Curtis dissimilarity data (**C**). The scatter plots show principal coordinate 1 (PC1) vs. principal coordinate 2 (PC2) with percentages of variation explained by the components indicated. The points are colored by the treatment group and time point.

To visually identify whether groups of samples cluster based on similarity to each other, PCoA plots were generated to highlight separation of groups of samples for unweighted UniFrac distance, weighted UniFrac distance, and Bray–Curtis dissimilarity distance (Figure 4). No statistically significant differences in microbial diversity between or within the placebo and synbiotic group at the baseline and end of trial were observed.

### 3.3. Associations between Gut Microbiota, Body Composition and Metabolic Parameters

In order to explore associations between the gut microbial species, body composition and metabolic parameters, regression and correlation analyses were performed as described in the Methods Section. Regression analysis to correlate relative microbial abundance of species present in the synbiotic supplement with body composition parameters and biomarkers of obesity found association between a decrease over time in blood glucose and an increase in *Lactobacillus* abundance in the synbiotic and placebo groups. In both groups combined, a mean decrease in HbA1C% (5.85%, see Table 1) was accompanied by a mean increase in *Lactobacillus* abundance (24.1-fold, see Figure 2; *p* = 0.044). However (and somewhat paradoxically), a decrease over time in body mass, BMI, waist circumstance, and body fat mass was associated with a statistically significant decrease in *Bifidobacterium* abundance in both the placebo and synbiotic groups (Table 4).

**Figure 5:**
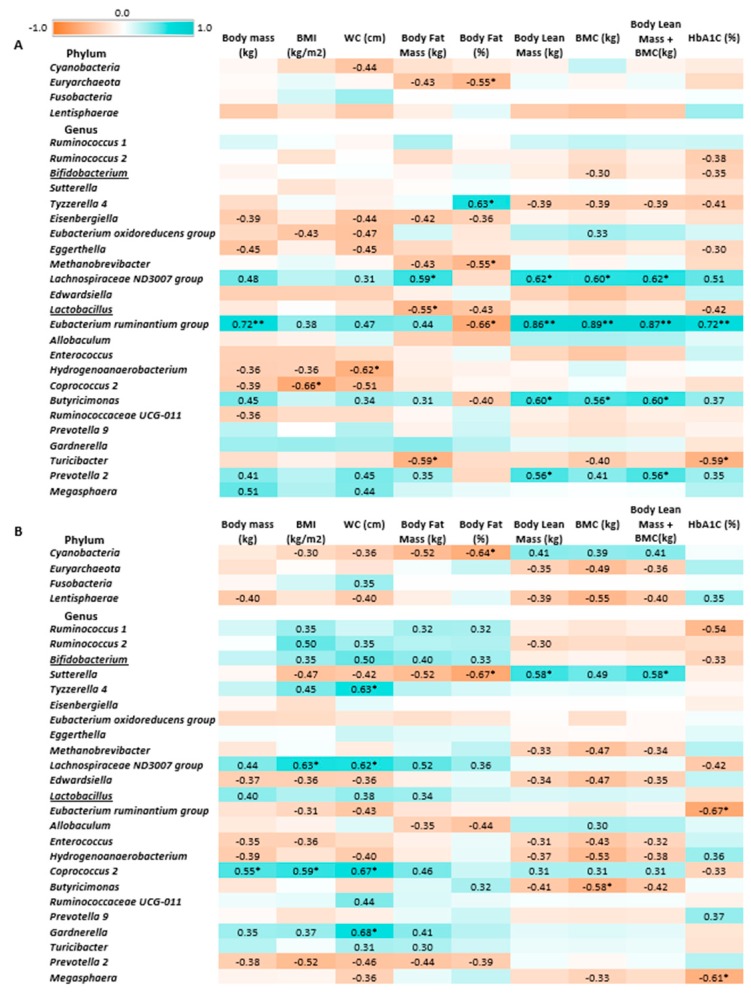
Heatmap of associations between gut microbiota, body composition and metabolic parameters in the placebo (**A**) and synbiotic groups (**B**) at the end of trial. *r* values were calculated using Pearson’s linear correlation test; * *p* < 0.05, ** *p* < 0.01. Pearson’s *r* values below 0.30 or above −0.30 are not indicated. Red-brown color indicates negative correlation, blue-green color—positive correlation.

The Pearson’s linear correlation test (Figure 5) did not indicate statistically significant associations between *Bifidobacterium* and *Lactobacillus* abundance and body composition parameters in the synbiotic group at the end of trial. A negatively correlated trend was observed between *Bifidobacterium* abundance and HbA1C levels in the synbiotic and placebo groups, whereas a positively correlated trend between *Bifidobacterium* abundance and, to a lesser extent, *Lactobacillus* abundance was observed with BMI, WC, and body fat mass in the synbiotic group. Interestingly, in the placebo group, *Lactobacillus* abundance was negatively correlated with body fat mass.

*Cyanobacteria, Sutterella, Butyricimonas,* and *Eubacterium ruminantium* abundance (which were increased following the synbiotic intervention) were significantly negatively correlated with body fat mass, and *Cyanobacteria* and *Sutterella* abundance was negatively correlated with body fat percentage. Additionally, *Butyricimonas* abundance positively correlated with BMC. *Eubacterium* abundance positively correlated with HbA1C percentage, whereas *Megasphaera* abundance (which was decreased after the synbiotic intervention) was negatively correlated with this marker. Positive correlations were found between *Coprococcus* abundance and body mass, BMI, and WC; *Lachnospiraceae* abundance and BMI, WC, and body fat mass; *Tyzzerella* and *Gardnerella* abundance and WC. 

Our data confirm several previously reported associations [31,32,33]; however, correlations found for *Lactobacillus* and *Bifidobacterium* were somewhat unexpected, although appeared to be promising for associations with blood glucose levels. The results obtained support the conclusion that the synbiotic supplement used in this intervention trial modulated the microbiota by increasing the abundance of the microbial genera associated with beneficial effects. Furthermore, these microbial changes may be associated with positive effects on metabolic parameters (blood glucose) in obesity. 

## 4. Discussion

This study was a placebo-controlled intervention clinical trial designed to examine the effects of a combination of probiotic bacteria *L. acidophilus*, *B. lactis, B. longum, B. bifidum* and a prebiotic mixture of galactooligosaccharides on the human gut microbiota in relation to changes in body composition and metabolic biomarkers in obese human subjects enrolled on a weight loss program. The weight loss program was a high-protein, low-carbohydrate, energy-restricted eating plan. Previous limited studies conducted using *L. acidophilus* and *B. lactis* have found that these probiotic species can be associated with decreased body weight and body fat percentage [34], while prebiotic galactooligosaccharides have been shown to improve markers of metabolic syndrome and modulate the gut microbiota and immune function in overweight adults [25,35,36]. However, this study focused on evaluating the effects of synbiotic supplementation in obesity during weight loss intervention.

The study has confirmed that a high-protein, low-carbohydrate, restricted-energy diet can be effectively used for weight loss in obese individuals. However, microbial breakdown of proteins within the large intestine has been associated with the production of genotoxic and cancer associated metabolites, e.g., *N*-nitroso compounds and ammonia [16]. As such, altering the gut microbiota community to one that is less proteolytic through the introduction of a synbiotic could be of benefit to the host. Previous evidence has shown that synbiotic supplementation contributes to altering microbial composition, resulting in benefits to weight loss and maintenance [37]. In the current study, the dietary changes were sufficient to elicit large changes to anthropometric parameters in both groups; however, synbiotic supplementation resulted in microbial changes that have frequently been associated with benefits to host health. How those changes relate to metabolic parameters remains to be elucidated.

A combination of the four strains of *Bifidobacterium* and *Lactobacillus acidophilus* and galactooligosaccharides in the synbiotic supplement resulted in a significant increase in abundance of these probiotic genera in the gut after a 3-month intervention. *Bifidobacterium* is largely considered a beneficial member of the microbial community and furthermore, this genus has been some association with anti-obesity effects [34,38,39]. In addition to this observation, further modulation of the gut microbiota was observed, for example, *Prevotella* and *Gardnerella* genera were significantly decreased after the synbiotic intervention (see Figure 2). Previous studies have reported that these genera are associated with chronic inflammatory conditions and positively correlated with obesity [40,41,42]. Therefore, the reduction in these genera could help to modulate the balance to improve metabolism within the host. Special caution is warranted when analyzing the data referring to *Prevotella*, a complex genus linked both to health and disease and, possibly, influenced by race/ethnicity [43]. However, statistically significant differences in the community composition of gut microbiota between groups (synbiotic vs. placebo) and time points (end vs. beginning of trial) using parameters of alpha-diversity (see Table 2) and beta-diversity (see Table 3) were not observed. Our data are compatible with a recent study that did not find a relationship between severe caloric restriction and changes in alpha-diversity [44]. In addition, correlation and regression analyses did not indicate statistically significant or apparently beneficial associations between genera contained in the synbiotic supplement (*Bifidobacterium* and *Lactobacillus*) and body composition parameters, including at the end of synbiotic intervention (see Figure 5B). Interestingly, the changes over time in body mass, BMI, waist circumstance, and body fat mass demonstrated a positive correlation trend with *Bifidobacterium* abundance in the synbiotic group, while changes in body fat mass were negatively correlated with *Lactobacillus* abundance in the placebo group. However, positive associations between relative abundance of *Bifidobacterium* and several body composition parameters appear to point to the unfavorable role of these bacteria in promoting weight loss, although potential benefits of this genera could me masked by the high-protein diet used in the study. High protein intake induces proteolytic fermentation in the gut with synthesis of compounds that have been implicated in the development of obesity and metabolic syndrome and modulating the gut microbiota [12,13,14,45] and the production of toxic metabolites [46]. Several studies have also found that increases in *Bifidobacterium* and *Lactobacillus* abundance is correlated with both pro- and anti-obesity effects in obese human subjects [47,48], thus complicating interpretation of the results. Individual differences in energy extraction may contribute to explain the observed differences [49]. Additionally, *Bifidobacterium* has been linked with improved barrier function in overweight individuals, thus adding a potential beneficial mechanism of action [50]. Therefore, more studies are needed to fully understand the observed divergences. 

A regression analysis performed to correlate microbial abundance of species contained in the synbiotic supplement with biomarkers of obesity found a novel significant association between a decrease over time in HbA1C percentage and an increase in *Lactobacillus* abundance, particularly in the synbiotic group. This is an important observation because it demonstrates a beneficial effect of increasing *Lactobacillus* abundance on potentially reducing blood glucose levels. Negative associations between *Megasphaera* abundance and *Eubacterium ruminantium* abundance with HbA1C levels were observed in the synbiotic group at the end of trial. *Eubacterium ruminantium* are xylanolytic bacteria (i.e., producing xylanase following dietary fiber fermentation) and *Megasphaera* bacteria utilize lactate [51], which can underlie the potential relationship of these species to decreasing blood glucose levels [52,53]. However, within the trial following the synbiotic, a decrease in *Megasphera* was observed, as well as an increase in *Eubacterium ruminantium*. This could imply that the synbiotic intervention and associated microbial changes could be linked to maintaining a normal blood glucose levels in obesity.

It should be also considered that the microbial shifts observed in this study may be associated with a positive impact on microbial fermentation within the large intestine. The microbial changes observed following synbiotic intervention included an increase in *Ruminococcus*, a genus known to produce butyrate. Butyrate, a short chain fatty acid (SCFA) that provides an energy source for the colonocytes and a histone deacetylase inhibitor, was linked to anti-cancer effects, thus providing protection against toxic metabolites that are produced on a high protein diet. SCFAs have also been recently associated with protection against type 1 diabetes [54]. In addition to this, *Lactobacillus* and *Bifidobacterium* are associated with positive effects of colonic health and, following the synbiotic intervention, have been associated with reducing fecal water genotoxicty, which is considered a biomarker for colon cancer [55]. Therefore, within the weight-loss diet employed, while the synbiotic treatment may have not had an additional impact on weight-loss parameters, it is possible that the changes in the gut microbiota could help to reduce detriments associated with a high-protein diet. 

It is important to emphasize that the present study was a randomized placebo-controlled intervention clinical trial and that analysis of the community composition of the gut microbiota between the treatment groups and time points was performed using comprehensive microbiome analysis, including alpha- and beta-diversity metrics and multivariate analysis of variance. The design of the study has allowed us to detect important novel associations between composition of the gut microbiota and metabolic parameters in obesity in the relatively limited number of participants in this clinical trial.

The results obtained and bioinformatic analysis support the conclusion that weight loss in human subjects participating in a high-protein, low-carbohydrate, energy-restricted eating weight loss program is accompanied by changes in gut microbiota that can be associated with increased genotoxicity [16]. Whilst the study conducted was small, our data support that the synbiotic used in this study modulated the human gut microbiota by increasing abundance of the microbial species that can be considered to be of benefit to their host and may help to counteract microbial fermentation associated with a high-protein diet whilst the role of these changes in relation to metabolic parameters requires more research. 

## Figures and Tables

**Table 1:** Characteristics of study participants at the beginning and end of the intervention clinical trial. BMI, Body Mass Index; WC, Waist Circumference; BMC, Bone Mineral Content; HbA1C, glycated hemoglobin. The duration of the trial was 3 months. The study enrollment period was 6 months, and subjects were assigned to the groups in a chronological order. n = 10 for the placebo group and n = 10 for the synbiotic group (7 subjects in the placebo group and 8 in the synbiotic group completed DXA scans). The results are expressed as mean ± SD. A one-way ANOVA with independent *t*-test was used for the group comparison (SPSS Statistics, v. 25). (*), *p* < 0.05, as compared between the beginning (baseline) and end of trial for the same group (placebo or synbiotic); *p* value, as compared between the placebo and synbiotic groups.

Characteristics/Parameters	Placebo	Synbiotic	*p*
**Sex** (%)			
Male	20.0	30.0	
Female	80.0	70.0	
**Age** (years)	47.0 ± 15.4	47.8 ± 8.99	0.88
**Height** (cm)	171.8 ± 12.9	163.4 ± 9.63	0.30
**Body mass** (kg)			
Baseline	97.6 ± 23.1	90.6 ± 11.9	0.40
End of trial	90.0 ± 21.9	83.4 ± 11.4	0.41
Body mass change (%)	7.78 ± 5.30 *	7.94 ± 3.88 *	0.86
**BMI** (kg/m^2^)			
Baseline	32.77 ± 4.51	34.20 ± 5.60	0.53
End of trial	30.14 ± 4.04	31.48 ± 5.23	0.53
BMI change (%)	8.02 ± 1.65 *	7.95 ± 1.52 *	0.82
**WC** (cm)			
Baseline	106.9 ± 12.47	109.6 ± 8.07	0.57
End of trial	101.1 ± 12.89	102.6 ± 8.48	0.76
WC change (%)	5.42 ± 5.78 *	6.38 ± 4.16 *	0.29
**Body Fat Mass** (kg)			
Baseline	40.66 ± 6.92	36.97 ± 11.35	0.47
End of trial	37.44 ± 6.99	34.06 ± 11.58	0.51
Fat mass change (%)	7.91 ± 2.73 *	7.87 ± 3.94 *	0.37
**Body Fat** (%)			
Baseline	40.97 ± 5.02	40.51 ± 8.96	0.90
End of trial	39.51 ± 4.53	39.13 ± 9.47	0.92
Body fat change (%)	3.56 ± 1.49 *	3.40 ± 2.97	0.20
**Body Lean Mass** (kg)			
Baseline	57.39 ± 17.76	51.13 ± 8.87	0.39
End of trial	55.61 ± 16.15	49.47 ± 8.64	0.36
Lean mass change (%)	3.10 ± 2.10 *	3.24 ± 1.14 *	0.25
**BMC** (kg)			
Baseline	2.66 ± 0.64	2.38 ± 0.48	0.34
End of trial	2.68 ± 0.67	2.38 ± 0.48	0.32
BMC change (%)	0.75 ± 0.05	0.16 ± 0.01	0.10
**Body Lean Mass + BMC** (kg)			
Baseline	60.05 ± 18.38	53.52 ± 9.35	0.39
End of trial	58.30 ± 16.78	51.86 ± 9.11	0.36
Lean mass + BMC change (%)	2.91 ± 2.08 *	3.10 ± 1.13 *	0.26
**HbA1C** (%)			
Baseline	5.36 ± 1.07	5.39 ± 0.28	0.93
End of trial	5.06 ± 0.37	5.06 ± 0.43	1.00
HbA1c change (%)	5.59 ± 0.89	6.12 ± 0.47 *	0.24

**Table 2:** Measuring statistical differences in alpha diversity between groups. Three alpha diversity metrics were used (Shannon Index, Chao1 Estimator, and Observed Species/OTUs). Significant differences between groups were determined using the estimated marginal means analysis applied to linear mixed model, which was built with alpha diversity as the response variable, the treatment group and time points as predictor variables, and subject number as a random variable.

Groups		Shannon Index		Chao1 Diversity		Observed Species	
Within	Between	Estimate	*p* Value	Estimate	*p* Value	Estimate	*p* Value
Baseline	Placebo—Synbiotic	0.144	0.643	295	0.359	222	0.388
End of Trial	Placebo—Synbiotic	0.145	0.641	205.4	0.521	180.9	0.481
Placebo	Baseline–End of Trial	0.208	0.208	76.94	0.577	76.2	0.46
Synbiotic	Baseline–End of Trial	0.209	0.206	-12.65	0.927	35.1	0.732

**Table 3:** Measuring statistical significance of beta diversity differences between groups using Permutational Multivariate Analysis of Variance (PerMANOVA) on models with beta diversity as the response variable, and treatment group and time point as predictive variables. Three beta diversity metrics were used (Bray–Curtis, weighted UniFrac, and unweighted UniFrac).

Groups		Bray-Curtis Dissimilarity		Weighted UniFrac		Unweighted UniFrac	
Within	Between	F-model	*p* Value	F-model	*p* Value	F-model	*p* Value
Baseline	Placebo—Synbiotic	1.393	0.133	0.84	0.516	1.155	0.232
End of Trial	Placebo—Synbiotic	1.389	0.158	0.923	0.379	1.038	0.325
Placebo	Baseline–End of Trial	0.376	0.996	0.389	0.932	0.351	1
Synbiotic	Baseline–End of Trial	0.431	0.983	0.305	0.958	0.392	1

**Table 4:** Association between changes over time in (body composition and metabolic parameters) and changes in gut microbiota abundance in the synbiotic and placebo groups (both groups combined). BMI, Body Mass Index; WC, Waist Circumference; HbA1C, glycated hemoglobin. Data were generated by applying analysis of variance to a mixed linear model, built with the abundance of a given microbe as the response variable, and body composition, metabolic parameter, treatment groups and time points as the predictor variables, with subject number as random variable.

Parameters	Change	Gut Microbiota	Change	*p*
HbA1C%	↓ 5.85%	*Lactobacillus*	↑ 24.1-fold	0.044
Body mass (kg)	↓ 7.86%	*Bifidobacterium*	↑ 263.8-fold	0.052
BMI (kg/m^2^)	↓ 7.98%	*Bifidobacterium*	↑ 263.8-fold	0.009
WC (cm)	↓ 5.90%	*Bifidobacterium*	↑ 263.8-fold	0.023
Body Fat Mass (kg)	↓ 7.89%	*Bifidobacterium*	↑ 263.8-fold	0.011

## Supplementary Materials

The following are available online at https://www.mdpi.com/2072-6643/12/1/222/s1, Figure S1: Sequence counts per sample for raw, filtered and classified sequences ordered by increasing classified sequence counts. Dashed gold line indicates the 25,000-sequence threshold used for rarefaction. A and B before sample numbers indicate the synbiotic and placebo groups, respectively, Figure S2: Mean relative abundance of species (%) by the treatment groups and time points, Figure S3: Relative abundance of phyla (S3A), genera (S3B) and species (S3C) per individual sample. A and B before sample numbers indicate the synbiotic and placebo groups, respectively, Table S1: Sequence quality metrics per sequencing run. % ≥ Q30: The proportion of base calls that have a confidence score of 30 or more. This is a commonly cited metric that can be used to evaluate the overall quality of a sequencing run. *Cluster Density*: How efficiently the sequencer is able to bind sequences of DNA to the flow cell; a higher density represents a more efficient sequencing run. *Clusters Passing Filter*: The proportion of clusters that meet the sequencer’s minimum quality threshold for sequence quality. Only clusters that pass filter are included in the sequencer’s FASTQ output. *Sequencing Yield*: Refers to how many nucleotide base pairs were called by the sequencer. 1 Gbp (Gigabase pair) means the sequencer generated 1 billion base pairs of output*. PhiX Alignment*: PhiX is a sequencing library that is used as a positive control on each sequencing run. The sequencer aligns reads to the PhiX library to calculate sequence-based quality control metrics. The requirement was that the percentage of reads that align to the PhiX library is within 20% of the spike-in amount of PhiX, Table S2: Software versions used for data analysis, Table S3: Body composition parameters, including DXA scans data. *A* and *B* before sample numbers indicate the synbiotic and placebo groups, respectively, Table S4: Complete OTU table. *A* and *B* before sample numbers indicate the synbiotic and placebo groups, respectively. See *Methods* sections for a description of classification, Table S5: Bonferroni adjusted *P* values.
