# Supplementary material for: Effects of Synbiotic Supplement on Human Gut Microbiota, Body Composition and Weight Loss in Obesity
Source: Nutrients. 2020 Jan 15;12(1):222. doi: 10.3390/nu12010222 (PMC7019807; doi:10.3390/nu12010222)
Supplement: Supplementary file 1 [file nutrients-12-00222-s001.zip › Supplemental Materials/Supplemental Figure 3B.pdf]

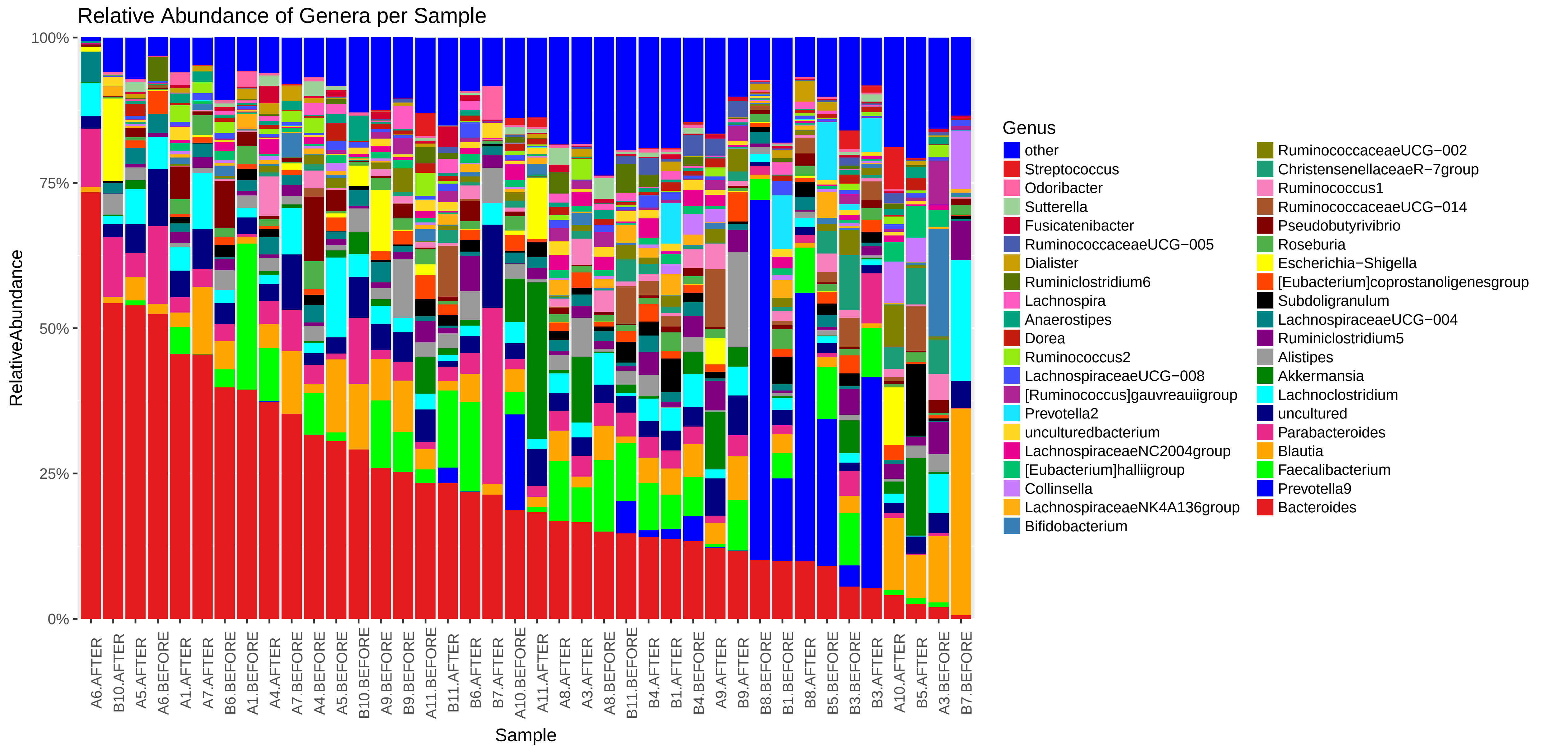

Relative Abundance of Genera per Sample
RelativeAbundance
100%
75%
50%
25%
0%
A6.AFTER
B10.AFTER
A5.AFTER
A6.BEFORE
A1.AFTER
A7.AFTER
B6.BEFORE
A1.BEFORE
A4.AFTER
A7.BEFORE
A4.BEFORE
A5.BEFORE
B10.BEFORE
A9.BEFORE
B9.BEFORE
A11.BEFORE
B11.AFTER
B6.AFTER
B7.AFTER
A10.BEFORE
A11.AFTER
A8.AFTER
A3.AFTER
A8.BEFORE
B11.BEFORE
B4.AFTER
B1.AFTER
B4.BEFORE
A9.AFTER
B9.AFTER
B8.BEFORE
B1.BEFORE
B8.AFTER
B5.BEFORE
B3.BEFORE
B3.AFTER
A10.AFTER
B5.AFTER
A3.BEFORE
B7.BEFORE
Sample
Genus
other
Streptococcus
Odoribacter
Sutterella
Fusicatenibacter
RuminococcaceaeUCG−005
Dialister
Ruminiclostridium6
Lachnospira
Anaerostipes
Dorea
Ruminococcus2
LachnospiraceaeUCG−008
[Ruminococcus]gauvreauiigroup
Prevotella2
unculturedbacterium
LachnospiraceaeNC2004group
[Eubacterium]halliigroup
Collinsella
LachnospiraceaeNK4A136group
Bifidobacterium
RuminococcaceaeUCG−002
ChristensenellaceaeR−7group
Ruminococcus1
RuminococcaceaeUCG−014
Pseudobutyrivibrio
Roseburia
Escherichia−Shigella
[Eubacterium]coprostanoligenesgroup
Subdoligranulum
LachnospiraceaeUCG−004
Ruminiclostridium5
Alistipes
Akkermansia
Lachnoclostridium
uncultured
Parabacteroides
Blautia
Faecalibacterium
Prevotella9
Bacteroides
