# Supplementary material for: Effects of Synbiotic Supplement on Human Gut Microbiota, Body Composition and Weight Loss in Obesity
Source: Nutrients. 2020 Jan 15;12(1):222. doi: 10.3390/nu12010222 (PMC7019807; doi:10.3390/nu12010222)
Supplement: Supplementary file 1 [file nutrients-12-00222-s001.zip › Supplemental Materials/Supplemental Figure S1.pdf]

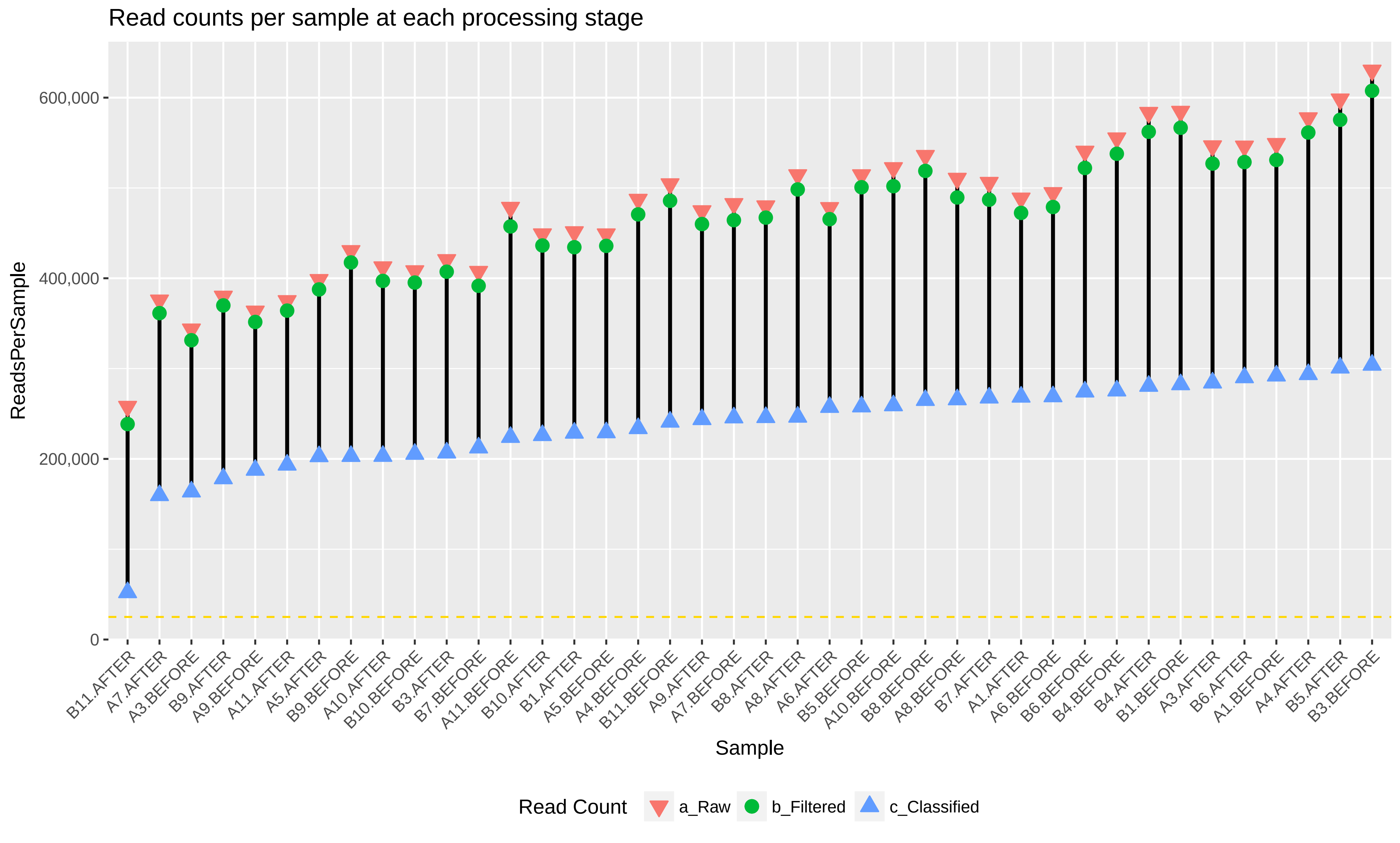
Read counts per sample at each processing stage
ReadsPerSample
600,000
400,000
200,000
0
B11.AFTER
A7.AFTER
A3.BEFORE
B9.AFTER
A9.BEFORE
A11.AFTER
A5.AFTER
B9.BEFORE
A10.AFTER
B10.BEFORE
B3.AFTER
B7.BEFORE
A11.BEFORE
B10.AFTER
B1.AFTER
A5.BEFORE
A4.BEFORE
B11.BEFORE
A9.AFTER
A7.BEFORE
B8.AFTER
A8.AFTER
A6.AFTER
B5.BEFORE
A10.BEFORE
B8.BEFORE
A8.BEFORE
B7.AFTER
A1.AFTER
A6.BEFORE
B6.BEFORE
B4.BEFORE
B4.AFTER
B1.BEFORE
A3.AFTER
B6.AFTER
A1.BEFORE
A4.AFTER
B5.AFTER
B3.BEFORE
Sample
Read Count
a_Raw
b_Filtered
c_Classified
