# Supplementary material for: Effects of Synbiotic Supplement on Human Gut Microbiota, Body Composition and Weight Loss in Obesity
Source: Nutrients. 2020 Jan 15;12(1):222. doi: 10.3390/nu12010222 (PMC7019807; doi:10.3390/nu12010222)
Supplement: Supplementary file 1 [file nutrients-12-00222-s001.zip › Supplemental Materials/Supplemental Table S1.docx]

| **Metric** | **Minimum Requirement** | **Results** |
| --- | --- | --- |
| % ≥ Q30 | ≥ 75% | 82.38 % |
| Cluster Density | ≥ 800 k/mm^2^ | 1,111 ± 19 k/mm^2^ |
| Clusters Passing Filter (%) | ≥ 85% | 86.49 ± 1.34 % |
| Sequencing Yield | ≥ 13.2 Gbp | 14.04 Gbp |
| PhiX Alignment (%) | 12% - 18% | 11.63 % |

**Table S1.** Sequence quality metrics per sequencing run.

*% ≥ Q30*: The proportion of base calls that have a confidence score of 30 or more. This is a commonly cited metric that can be used to evaluate the overall quality of a sequencing run. *Cluster Density*: How efficiently the sequencer is able to bind sequences of DNA to the flow cell; a higher density represents a more efficient sequencing run. *Clusters Passing Filter*: The proportion of clusters that meet the sequencer’s minimum quality threshold for sequence quality. Only clusters that pass filter are included in the sequencer’s FASTQ output. *Sequencing Yield*: Refers to how many nucleotide base pairs were called by the sequencer. 1 Gbp (Gigabase pair) means the sequencer generated 1 billion base pairs of output*. PhiX Alignment*: PhiX is a sequencing library that is used as a positive control on each sequencing run. The sequencer aligns reads to the PhiX library to calculate sequence-based quality control metrics. The requirement was that the percentage of reads that align to the PhiX library is within 20% of the spike-in amount of PhiX.
