# Supplementary material for: Effects of Synbiotic Supplement on Human Gut Microbiota, Body Composition and Weight Loss in Obesity
Source: Nutrients. 2020 Jan 15;12(1):222. doi: 10.3390/nu12010222 (PMC7019807; doi:10.3390/nu12010222)
Supplement: Supplementary file 1 [file nutrients-12-00222-s001.zip › Supplemental Materials/Supplemental Table S2.docx]

| **Software** | **Version** |
| --- | --- |
| Trimmomatic | 0.36 |
| NINJA-OPS | 1.5.1 |
| SILVA taxonomic database | 123 |
| QIIME | 1.9.1 |
| SPSS Statistics | 25 |
| R | 3.3.2 |
| **R packages** |  |
| emmeans | 1.2 |
| lme4 | 1.1-17 |
| Matrix | 1.2-14 |
| scales | 0.5.0 |
| ggplot2 | 2.2.1 |
| reshape2 | 1.4.3 |
| made4 | 1.48.0 |
| scatterplot3d | 0.3-41 |
| gplots | 3.0.1 |
| RColorBrewer | 1.1-2 |
| ade4 | 1.7-11 |
| vegan | 2.5-1 |
| lattice | 0.20-35 |
| permute | 0.9-4 |

**Table S2.** Software versions used for data analysis.
