# Supplementary figures and images for: Effects of Synbiotic Supplement on Human Gut Microbiota, Body Composition and Weight Loss in Obesity
Source: Nutrients. 2020 Jan 15;12(1):222. doi: 10.3390/nu12010222 (PMC7019807; doi:10.3390/nu12010222)

Relative Abundance of Phyla per Sample

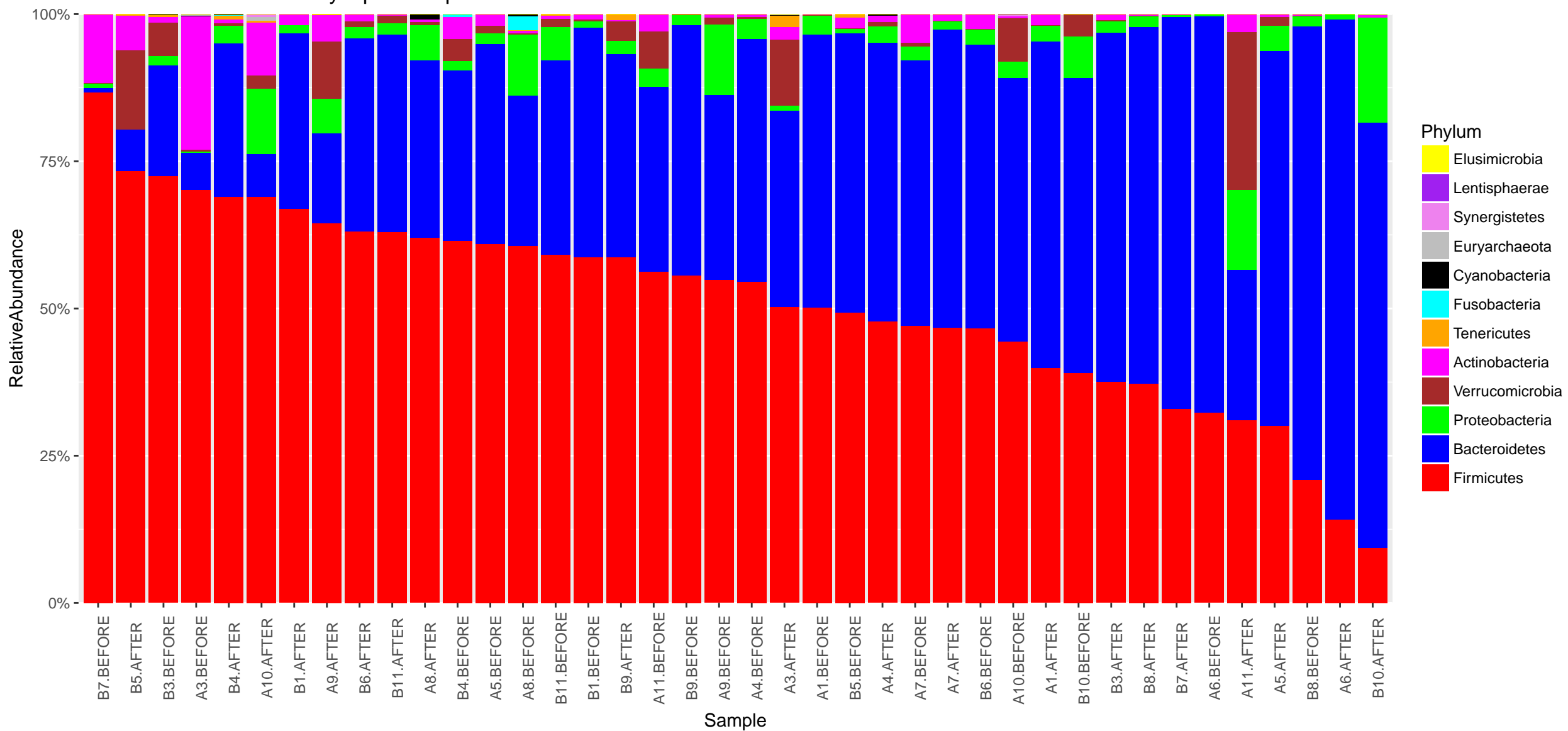

Supplement: Supplementary file 1 [file nutrients-12-00222-s001.zip › Supplemental Materials/Supplemental Figure 3A.pdf]

# Relative Abundance of Species per Sample

RelativeAbundance

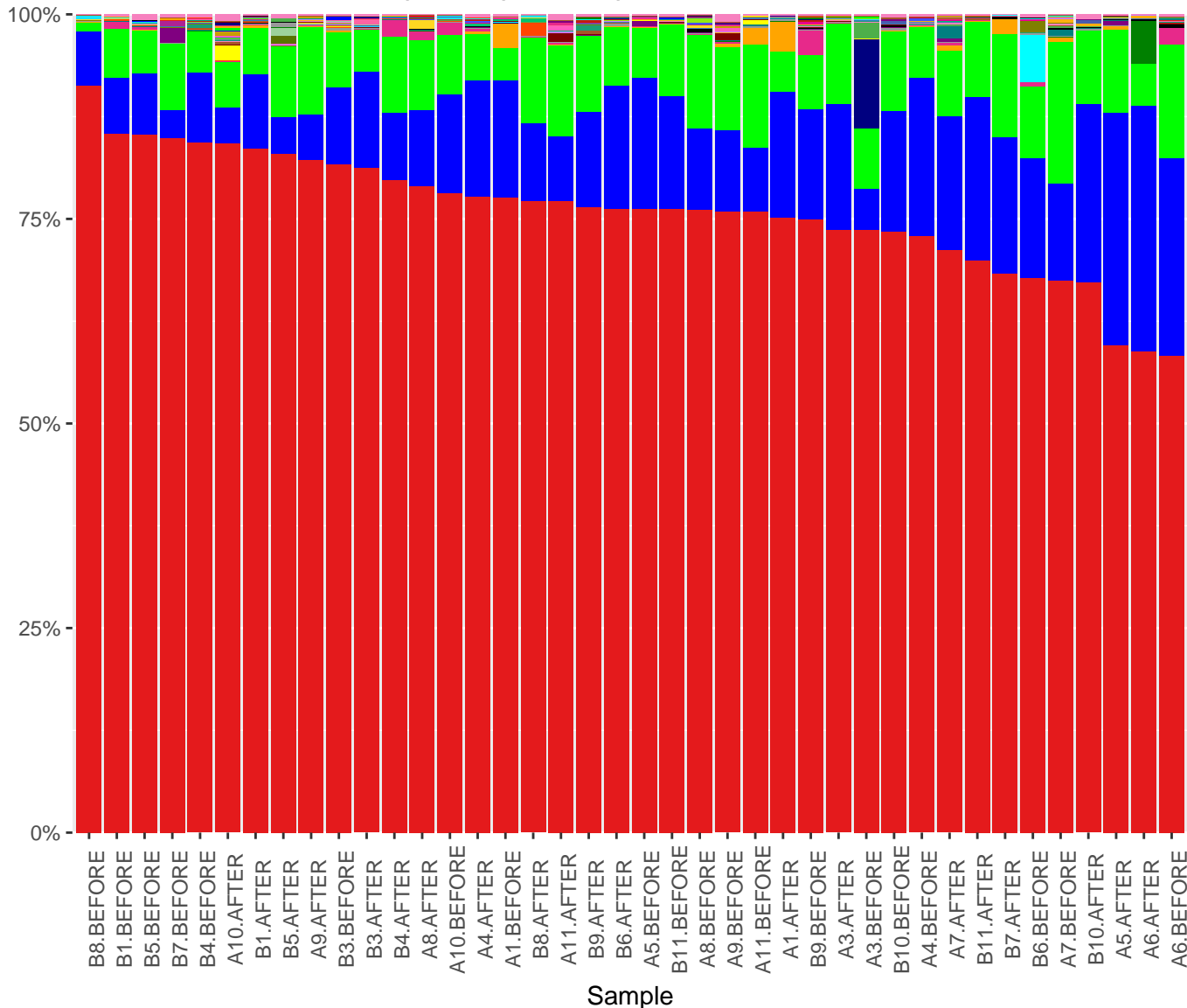

Supplement: Supplementary file 1 [file nutrients-12-00222-s001.zip › Supplemental Materials/Supplemental Figure 3C.pdf]

# Mean Relative Abundance of Species per Group

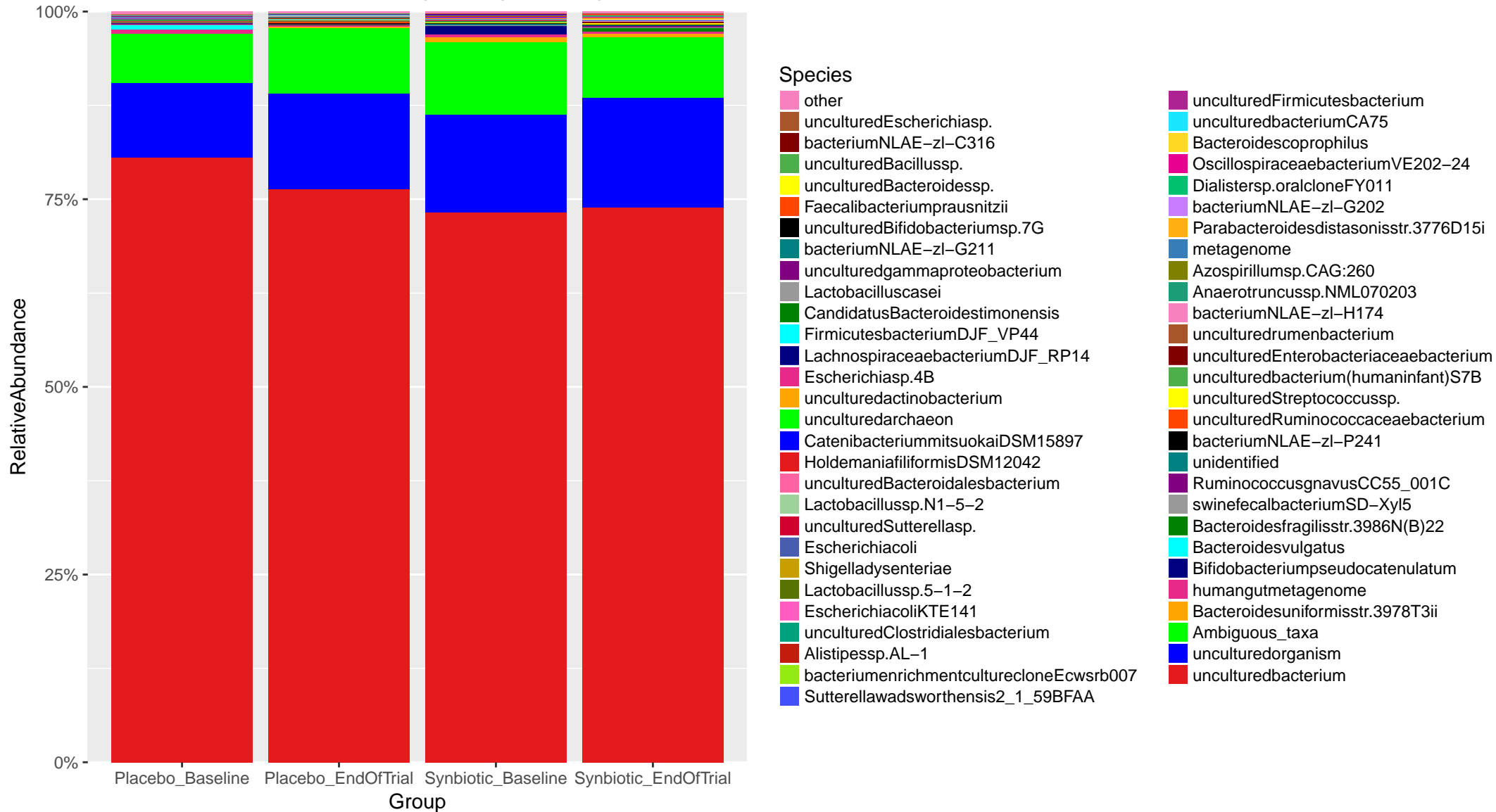

Supplement: Supplementary file 1 [file nutrients-12-00222-s001.zip › Supplemental Materials/Supplemental Figure S2.pdf]
